# MBTPS2 acts as a regulator of lipogenesis and cholesterol synthesis through SREBP signalling in prostate cancer

**DOI:** 10.1038/s41416-023-02237-7

**Published:** 2023-03-29

**Authors:** Amy J. Tibbo, Andrew Hartley, Richa Vasan, Robin Shaw, Laura Galbraith, Ernest Mui, Hing Y. Leung, Imran Ahmad

**Affiliations:** 1 School of Cancer Sciences, College of Medical, Veterinary and Life Sciences, University of Glasgow, Glasgow, G61 1QH UK; 2 CRUK Beatson Institute, Garscube Estate, Switchback Road, Bearsden, Glasgow, G61 1BD UK

**Keywords:** Prostate cancer, Cancer metabolism

## Abstract

**Background:**

Prostate cancer is the most common cancer in men in the developed world, with most deaths caused by advanced and metastatic disease which has no curative options. Here, we identified *Mbtps2* alteration to be associated with metastatic disease in an unbiased in vivo screen and demonstrated its regulation of fatty acid and cholesterol metabolism.

**Methods:**

The *Sleeping Beauty* transposon system was used to randomly alter gene expression in the *Pten*^*Null*^ murine prostate. MBTPS2 was knocked down by siRNA in LNCaP, DU145 and PC3 cell lines, which were then phenotypically investigated. RNA-Seq was performed on LNCaP cells lacking *MBTPS2*, and pathways validated by qPCR. Cholesterol metabolism was investigated by Filipin III staining.

**Results:**

*Mbtps2* was identified in our transposon-mediated in vivo screen to be associated with metastatic prostate cancer. Silencing of *MBTPS2* expression in LNCaP, DU145 and PC3 human prostate cancer cells reduced proliferation and colony forming growth in vitro. Knockdown of *MBTPS2* expression in LNCaP cells impaired cholesterol synthesis and uptake along with reduced expression of key regulators of fatty acid synthesis, namely *FASN* and *ACACA*.

**Conclusion:**

MBTPS2 is implicated in progressive prostate cancer and may mechanistically involve its effects on fatty acid and cholesterol metabolism.

## Background

Prostate cancer (PC) is the most common cancer in adult males in the developed world and the second leading cause of cancer deaths [1]. As the disease advances, androgen inhibition aside, there remains a paucity of targeted treatments [2]. It is this group of patients that has the greatest unmet need, and for which mechanistic insight, and development of new therapies are desperately sought.

Probasin-Cre-recombinase *Pten*^*fl/fl*^ *(*referred to thereafter as *Pten*^*Null*^*)* mice develop slow growing prostate tumours which rarely invade and metastasise [3]. By inducing somatic mutations to accelerate tumourigenesis, we can identify genes capable of driving locally advanced and metastatic PC. We have previously published on the use of the forward mutagenesis-based *Sleeping Beauty* (SB) system, which randomly alter gene expression upon the insertion of a transposon. Disruption of nearby tumour suppressor genes or overexpression of oncogenes may promote tumourigenesis, leading to locally advanced and/or metastatic PC, thus identifying candidate “driver” events [4]. One of the genes identified from this screen encodes for the protein MBTPS2 (membrane bound transcription factor peptidase site-2 protease, also known as S2P), which has previously published roles in cholesterol and fatty acid metabolism as well as in endoplasmic reticulum (ER) stress response [5–7].

Increased de novo fatty acids synthesis is a recognised hallmark of cancer [8]. Fatty acids are the essential components of membrane lipids as well as representing an important source of energy necessary for proliferating cells. Overexpression of many of the fatty acid and cholesterol synthesis genes have been implicated as oncogenic drivers of PC [9].

**Fig. 1:**
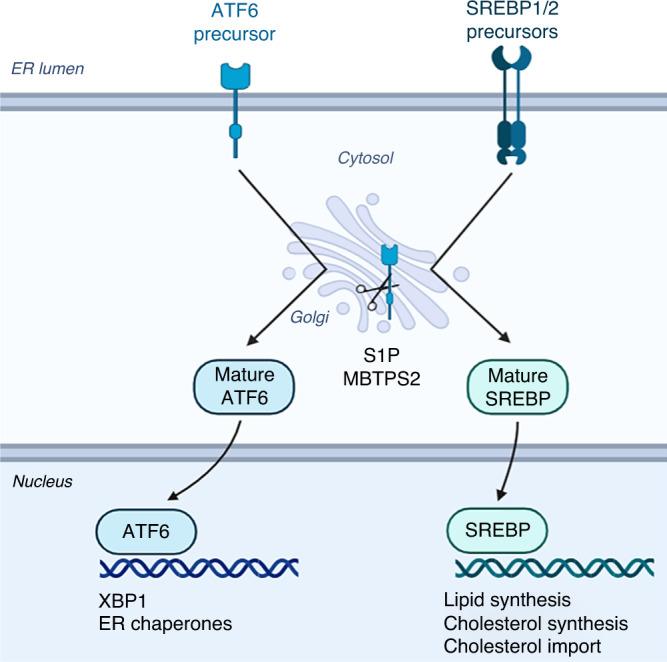
Diagrammatic representation of the ATF6 and SREBP activation by MBTPS2. ATF6 and SREBP are synthesised in their inactive precursor forms and are embedded in the endoplasmic reticulum. ER stress and lipid/cholesterol deprivation can trigger the release of ATF6 and SREBPs, respectively. These inactive proteins are trafficked to the Golgi where they are sequentially cleaved by S1P and MBTPS2. Cleavage results in the release of mature, transcriptionally active proteins which are then shuttled to the nucleus. Here, ATF6 can bind and activate expression of ER-stress response genes, whereas SREBP activate expression of lipid and cholesterol metabolism.

Regulated intramembrane proteolysis (RIP) plays an integral role in maintaining multiple cellular pathways [10]. The most well described RIP pathway is carried out by serine proteases, S1P (site-1 protease) and MBTPS2. The sequential cleavage of membrane spanning proteins results in the release of a mature N-terminal fragments that can shuttle to the nucleus and function as transcription factors. Among reported S1P and MBTPS2 targets are the sterol regulatory element binding proteins (SREBPs) and the activating transcription factor 6 (ATF6) [5–7]. After sequential cleavage of SREBPs by S1P and MBTPS2 (Fig. 1), mature SREBP traffics to the nucleus where it binds to sterol regulatory element sequences in the promoters of multiple target genes involved in de novo lipogenesis and cholesterol biosynthesis [11]. Individual SREBP isoforms control the expression of distinct sets of target genes. Both SREBP-1a and SREBP-1c activate the transcription of genes involved in the synthesis of fatty acids and triglycerides, including Fatty Acid Synthase (*FASN*) and Stearoyl-CoA desaturase-1 (*SCD-1*). SREBP2 predominantly controls genes necessary for cholesterol uptake and biosynthesis, including low density-lipoprotein receptor (LDLR), HMG CoA reductase and HMG CoA synthase.

In this study we present data from our in vivo screen implicating *MBTPS2* in progressive PC, and demonstrate how MBTPS2 regulates SREBP target genes to control lipogenesis and cholesterol biosynthesis pathways, both of which are important drivers in human PC.

## Methods

### Murine samples

Murine samples were collected as previously described [4]. One prostate sample was used per mouse. Mice were bred to be Probasin-Cre Recombinase positive and *Pten*^*flox/flox*^ as control or Probasin-Cre Recombinase positive and *Pten*^*flox/flox*^ with the *Sleeping Beauty* system (*T2/Onc3*^*het*^*Rosa*^*26Lox66SBLox71/+*^).

### Cell culture

LNCaP, DU145 and PC3 cell lines were maintained in RPMI-1640 medium (Sigma–Aldrich) containing 10% (v/v) Foetal Bovine Serum (FBS) (Gibco) and 1% L-Glutamine (Gibco) at 37 °C in 5% CO_2_ atmosphere. RWPE-1 cell line was maintained in Keratinocyte serum-free medium (Thermo Fisher) supplemented with human recombinant epidermal growth factor (rEGF) and bovine pituitary extract (BPE) as supplied, 10% FBS (Gibco), and 1% L-Glutamine (Gibco). Cell lines were authenticated and confirmed negative for mycoplasma by Molecular and Advanced Technologies service at the Cancer Research UK Beatson Institute.

### siRNA treatment

siRNAs were purchased from Dharmacon: ON-TARGETplus Human MBTPS2 SMARTPool; ON-TARGETplus Human SREBP1 SMARTPool; ON-TARGETplus Human SREBP2 SMARTPool and ON-TARGETplus non-targeting siRNA (NTS) (sequences targeted shown in Supplementary Table 1). Cell lines were reverse transfected with individual siRNAs, to a final concentration of 25 nM, using Lipofectamine RNAiMAX (Invitrogen) following the manufacturer’s protocols, three technical replicates per experiment. To assess siRNA knockdown efficiency, RNA was collected for quantitative real-time PCR (qPCR) analysis or cells were seeded onto coverslips for immunocytochemistry analysis.

### Cell growth analysis

Cell growth was assessed using the IncuCyte Zoom cell imager (Essen) according to manufacturer’s protocol. LNCaP, DU145 and PC3 cells were treated with 25 nM siRNA using RNAiMAX. Cells were removed from transfection medium and normal growth medium was added prior to being moved to the IncuCyte for imaging. Images were taken every 2 h for a total of 120 h. The IncuCyte Zoom 2018A software was trained to identify cells from each respective cell line and measure the % of confluency in each well. Cell confluency was normalised for each well at the 0-h time point and the relative change was calculated for each time point thereafter. Data presented in this study represents the mean relative confluency at each time point from three independent experiments. Each experiment included three technical replicates. In conjunction with the IncuCyte analysis, siRNA treated LNCaP, DU145 and PC3 cells were counted after 96 h of culturing using the CASY cell counter (OMNI Life Science). Counts were performed in three experimental replicates with two technical replicates carried out for each n number.

### Colony forming assay

LNCaP, DU145 and PC3 cells were reverse transfected with 25 nM of non-targeting siRNA (NTS) or MBTPS2 targeted siRNA and incubated for 24 h. Cells were then reseeded and left for a further 96 h to allow colonies to form. Cells were fixed and stained with Crystal Violet (0.5%w/v) and colonies were quantified fluorescent detection using the Odyssey system (Licor).

### Immunohistochemistry (IHC)

IHC was performed as described previously [4]. Antibody against Ki67 (VP-RM04; Vector Labs) was diluted 1:100 and IHC was performed following the manufacturer’s instructions by incubating with sample at 4 °C overnight. Citrate buffer and water bath antigen retrieval was used at 99 °C for 50 min.

### Immunocytochemistry

Prior to staining, LNCaP, PC3 and DU145 cells were seeded onto sterile coverslips and transfected with NTS siRNA, MBTPS2 targeted siRNA or SREBP targeted siRNA. After 24-h incubation, coverslips were fixed in 4% Paraformaldehyde for 1 h at room temperature. Coverslips were washed three times for 10 min with PBS and blocked for 1 h in blocking buffer (PBS supplemented with 0.5% BSA and 0.25% Triton X-100). Primary antibodies (MBTPS2, Cell Signalling #2157; FASN, Abcam #ab22759; SREBP1, BD-Biosciences #557036; SREBP2, BD Biosciences # 557037) were added at a 1:100 or 1:500 dilution in blocking buffer and incubated at 4 °C overnight in humidity chamber. After further PBS washes, Alexa-Fluor secondary antibodies (ThermoFisher, A32766 and A32734) were added to coverslips and incubated for 2 h at room temperature. Coverslips were washed a further three times for 10 min in PBS and mounted using ProLong™ Gold Antifade Mountant with DAPI (ThermoFisher, P36941) onto slides and allowed to dry. Imaging was carried out on the Ziess710 microscope using ZenBlack software and analysis performed using Fiji (ImageJ) software. Each experiment included three technical replicates.

### Filipin III cholesterol assay

LNCaP cells treated with siRNA targeted to SREBP or MBTPS2, and control groups treated with NTS were seeded onto coverslips and allowed to adhere prior to carrying out the Filipin III cell-based cholesterol assay (Abcam, ab133116) following the manufacturer’s protocol. Each experiment included two technical replicates.

### RNA extraction

Total mRNA was extracted from LNCaP, DU145 and PC3 cells after siRNA treatment using the RNeasy Mini Kit (QIAGEN) according to the manufacturer’s instructions including the on-column DNase treatment. Isolated RNA was quantified using the NanoDrop 2000 spectrophotometer (Thermo Fisher Scientific).

### Quantitative PCR

First-strand cDNA was synthesised by reverse transcription from extracted RNA samples using the High-Capacity cDNA Transcription kit (Applied Biosystems) following the manufacturer’s protocol. QPCR was carried out using TaqMan Universal Master Mix (Thermo Fisher Scientific) with primer appropriate Universal ProbeLibrary probes (Roche) and three technical replicates per sample. Taq-man QPCR was carried out as previously described [4]. The *CASC3* gene was used as the reference to normalise expression levels. Data regarding gene expression are shown relative to levels in control cells. (List of primers and universal probe number shown in Supplementary Table 2).

### RNA sequencing

RNA sequencing (RNA-Seq) was carried out as previously described [12]. Briefly, the quality of the RNA produced was tested using an Agilent 220 Tapestation on RNA screentape. Three independent experimental replicates of each sample with three technical replicates were sequenced for each experiment. Quality checks on the raw RNA-Seq data files were conducted by Mr William Clark (Core Sequencing Services, Beatson Institute) using FastQC [13].

Using an adapted method, libraries for cluster generation and DNA sequencing were prepared [14]. The libraries were run on Next Seq 500 using the High Output 75 cycles kit (2 × 36 cycles of paired end reads, single index). RNA-seq reads were aligned to the CRCh38 [15] version of the human genome using the tophat2 version 2.0.13 [16] with Bowtie version 2.4.4.0 [17]. Significant changes in expression levels were identified and investigated for statistical significance using a combination of HTseq version 0.9.1 [18] and the R 3.3.3 software accompanied by packages from the Bioconductor data analysis suite and DESeq2 [19].

### Statistical analysis

Statistical analyses, except for the RNA-seq data, were performed using GraphPad Prism v9.3.1. Testing comprised of unpaired two tailed *t*-tests, Mann–Whitney, Kaplan–Meier survival analysis and one- and two-way ANOVA with post tests for multiple comparisons (detailed in figure legends). All experiments were performed in experimental replicates, with technical replicates for each experiment noted. The graphs represent the mean data from the repeated experiment or sample ±SEM.

## Results

### Insertions of transposon in the *Mbtps2* gene are associated with accelerated prostate carcinogenesis in vivo

**Fig. 2:**
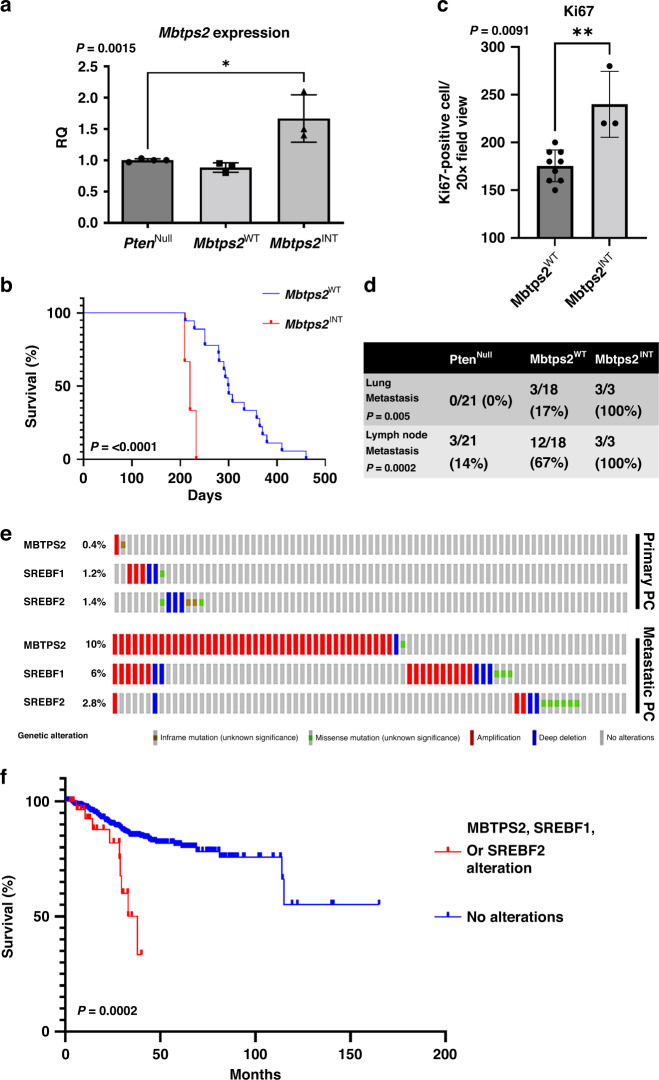
Identification of MBTPS2 by *Sleeping Beauty* transposon screen on a background of *Pten*^Null^-driven prostate cancer. **a** RT-PCR for *Mbtps1* expression in prostate tumours from *Pten*^*Null*^*, Mbpts2*^*WT*^ and *Mbpts2*^*INT*^ mice (*n* = 4 mice for *Pten*^*Null*^, *n* = 3 mice for *Mbtps2*^*WT*^ and *Mbtps2*^INT^, *p < 0.016; unpaired *t*-test). **b** Kaplan–Meier (log-rank) curve demonstrating reduced survival in the *Mbtps2*^*INT*^ (*n* = 3) mice in comparison to *Mbtps2*^*WT*^ (*n* = 18) mice, *P* < 0.0001; log-rank (Mantel–Cox) test. **c** Quantification of Ki67-positive cells in *Mbtps2*^*INT*^ prostate tumours (*n* = 3 mice, minimum cell count of 100 cells per mouse) demonstrated increased levels of proliferation compared with tissue from *Mbtps2*^*WT*^ tumours (*n* = 9, minimum cell count of 100 cells per mouse), **p = 0.0091; Mann–Whitney). **d** Percentages of mice with lung and lymph node metastases in *Mbtps2*^INT^ mice compared to *Mbtps2*^WT^ mice tested by Fisher’s exact test. **e** *MBTPS2, SREBF1* and *SREBF2* alterations visualised on CBioPortal using TCGA Firehose Legacy cohort for primary PC, and SU2C/PCF Dream Team(31) for the metastatic PC cohort. **f** Kaplan–Meier (log-rank) curve using TCGA Firehose Legacy cohort for primary PC, and SU2C/PCF Dream Team(31) showing patients with *MBTPS2, SREBF1* or *SREBF2* alterations (*N* = 31) and without these alterations (*N* = 594). *P* = 0.0002; log-rank (Mantel–Cox) test.

*Pten*^*Null*^ *(PB-Cre4:Pten*^*fl/fl*^*)* mice develop high-grade prostatic intraepithelial neoplasia (HGPIN) at 3 months of age, progressing to PC (>10 months) without evidence of metastasis at up to 18 months [3]. In *Pten*^*Null*^ mice with the forward mutagenesis-based *Sleeping Beauty* system (*SB:Pten*^*Null*^ (*PB-Cre4Pten*^*fl/fl*^*T2/Onc3*^*het*^*Rosa*^*26Lox66SBLox71/+*^))^,^ prostate carcinogenesis was accelerated, with a reduction in time to clinical endpoint (*SB:Pten*^*Null*^, median 293 days vs *Pten*^*Null*^ mice, median 469 days) [4]. Transposon insertion sites in tumours from *SB:Pten*^*Null*^ mice were identified, and in three mice, we identified a single insertion sites in the *Mbtps2* gene, resulting in an increase in transcript levels in their prostate tumours (Fig. 2a). SB:*Pten*^*Null*^ mice with insertions in *Mbtps2* (*Mbtps2*^*INT*^) had a significantly reduced survival rate compared to SB:*Pten*^*Null*^ mice without *Mbtps2* insertions (*Mbtps2*^*WT*^) (but harbouring insertions involving other genes) (median, 215 vs 299.5 days, respectively) (Fig. 2b). Prostate cancer in each of the *Pten*^*Null*^, *SB:Pten*^*Null*^ *Mbtps2*^*WT*^ and *SB:Pten*^*Null*^ *Mbtps2*^*INT*^ cohorts all progress to adenocarcinoma as representative models of advanced prostate cancer (Supplementary Fig. 1). Mice with the *Mbtps2*^*INT*^ demonstrated increased proliferation in their prostate tumours compared to those in the *Mbtps2*^*WT*^ group (Fig. 2c). Intriguingly, all three mice with *Mbtps2* alterations developed lung and lymph node metastases (Fig. 2d). In the SU2C/PCF Dream Team cohort, 10% of patients with metastatic PC have evidence of *MBTPS2* amplification, along with alterations in *SREBP1* (6%), and *SREBP2* (2.8%), relatively higher when compared to patients without metastasis (the TCGA Firehose Legacy cohort [20]) (Fig. 2e). Furthermore, patients with alterations in the *MBTPS2*, *SREBP1* or *SREBP2* genes (*N* = 31) had a significantly poorer survival compared to patients without these alterations (*N* = 594) (Fig. 2f). MBTPS2 is found on the X-chromosome, though amplification of MBTPS2 did not significantly co-occur with AR amplification (Supplementary Fig. 2). This suggests that MBTPS2 amplification is its own genomic event and is not merely a passenger event with AR amplification. This elevated amplification of MBTPS2 in metastatic PC is also seen across other PC cohorts on CBioPortal (Supplementary Fig. 3).

### *MBTPS2* knockdown decreases growth and survival in human prostate cancer cells

**Fig. 3:**
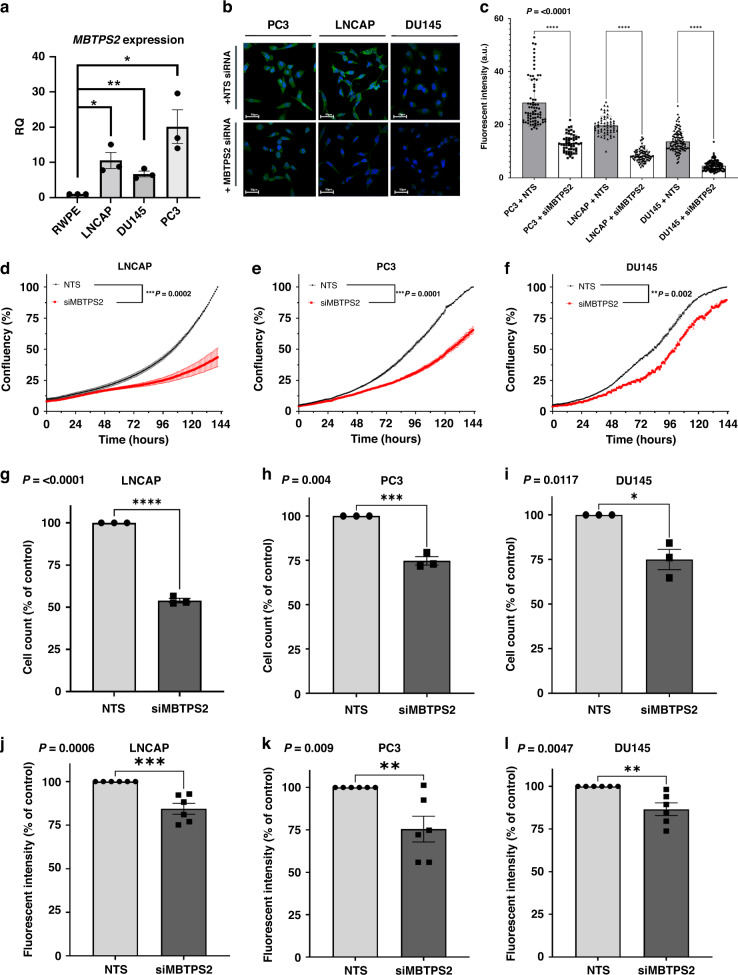
siRNA-mediated knockdown of MBTPS2 in human PC cells results in decreased growth and survival. **a** qPCR relative quantification (RQ) data reflecting the mRNA levels of *MBTPS2* in DU145, PC3 and LNCaP cell lines normalised to the level in RWPE cells (*n* = 3 independent experimental replicates, error bars are ±SEM, *p < 0.05, **p < 0.01 ANOVA with Tukey’s analysis). **b**, **c** Representative ICC imaging of MBTPS2 (green) after siRNA targeted knockdown of *MBTPS2* compared to non-targeting controls (*n* = 3 experiments, >40 cells imaged per technical replicate from each experiment, error bars are ±SEM, *P* < 0.0001, ANOVA with Tukey’s analysis). **d**–**f** Growth analysis data from LNCaP, PC3, and DU145 cells treated with siRNA targeted to *MBTPS2* (red) in comparison to non-targeting control (black) (*n* = 3 independent experiments, error bars are ±SEM, **P = 0.002, ***P < 0.001, one-way ANOVA). **g**–**i** Cells were counted at a time point of 96 h. Decreased cell numbers were shown in the siRNA-mediated knockdown of *MBTPS2* in LNCaP, PC3 and DU145 cells vs control cells treated with non-targeting siRNA (*n* = 3 independent experiments, error bars are ±SEM, *p < 0.05, ***P < 0.001, ****P < 0.0001, Mann–Whitney). **j**–**l** After siRNA-mediated knockdown, LNCaP, PC3 and DU145 cells were functionally assessed using a colony forming assay showing a reduction in MBTPS2 knockdown cells vs non-targeting control (*n* = 3 experiments with 2 technical replicates per experiment, error bars are ±SEM, **p < 0.01, ***p < 0.001, Mann–Whitney).

We next characterised *MBTPS2* mRNA expression in a panel of human PC cell lines: LNCaP, DU145 and PC3, along with the control benign RWPE-1 prostate epithelial cells. The cancer cell lines expressed *MBTPS2* mRNA at significantly higher levels than RWPE-1 cells (PC3 vs RWPE-1 ~20-fold change; LNCaP vs RWPE-1 10-fold change; and DU145 vs RWPE ~6-fold change) (Fig. 3a). The levels of MBTPS2 protein expression were further confirmed by immunocytochemistry, with both PC3 and LNCaP cells demonstrating higher MBTSP2 immunoreactivity, consistent with the mRNA data (Fig. 3b upper panel). Immunoreactivity was specific to MBTSP2 as the signals were reduced following siRNA-mediated knockdown of *MBTPS2*, consistently with ~50% reduction in MBTPS2 protein levels in all PC cell lines (Fig. 3b lower panel, Fig. 3c). siRNA-mediated gene knockdown of *MBTPS2* significantly decreased cell proliferation in all 3 cancer cell lines in growth assay over 120 h (Fig. 3d–f). Cell counts at 96 h confirmed that LNCaP, PC3 and DU145 growth was reduced by 46%, 25% and 25%, respectively, in comparison to NTS controls cells (Fig. 3g–i). Furthermore, a colony forming assay revealed significantly reduced growth in all three cell lines when MBTPS2 expression was silenced (LNCaP 16% reduction; PC3 25% reduction; DU145 13% reduction) (Fig. 2j–l).

Overexpression of MBTPS2 did not alter growth of LNCaP and surprisingly decreased growth in PC3 (Supplementary Fig. 4A-D). FASN expression was not changed in either cell line following MBTPS2 overexpression. As LNCaP and PC3 already have high basal *Mbtps2* expression in comparison to RWPE-1 (Fig. 3a), further increases in MBTPS2 expression may not be biologically relevant.

### Decrease of MBTPS2 leads to downregulation of cholesterol and lipid synthesis pathways

To further investigate the molecular impact of suppressing *MBTPS2* expression in PC we carried out transcriptomic analysis, selecting LNCaP cells as the cell model of choice based on their lack of PTEN expression (as in our *SB*:*Pten*^*Null*^ model) and the observed phenotypic changes on knockdown of *MBTPS2*. Principle component analysis (PCA) demonstrated that LNCaP cells cluster separately following knockdown of MBTPS2 compared to control cells (Supplementary Fig. 5A, B), with minimal variance between experimental replicates within each condition. Upon silencing *MBTPS2* expression, 681 genes were identified to be differentially expressed, 151 upregulated and 530 downregulated. (Supplementary Fig. 5C, D). Consistent with the hypothesis that MBTPS2 functions a positive regulator of gene expression via SREBP cleavage, knockdown of *MBTPS2* demonstrated a bias towards reducing gene expression (Supplementary Fig. 5C, D). The top downregulated networks (Supplementary Fig. 6B) were predominantly cellular responses to DNA damage and cell cycle progression suggesting that a reduction in MBTPS2 impairs growth signalling.

**Fig. 4:**
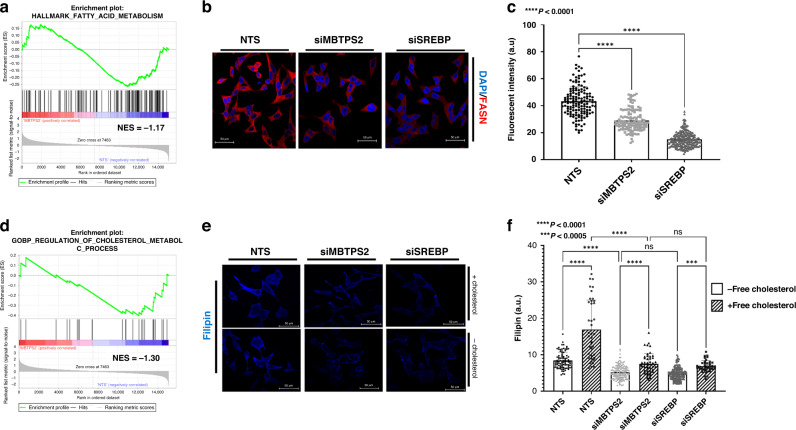
siRNA targeted knockdown of MBTPS2 downregulates the Cholesterol and Lipid synthesis pathways. **a** Gene set enrichment analysis of Hallmark fatty acid metabolism comparing LNCaP NTS vs LNCaP siMBTPS2 (*n* = 3 independent experiments, normalised enrichment score (NES) = −1.17). **b** ICC images representative of FASN staining after NTS, siMBTPS2 or siSREBP treatment in LNCaP cells (FASN shown in red, DAPI in blue). **c** Analysis of ICC imaging of FASN (*n* = 3 independent experiments with >40 cells imaged per experiment, error bars are ±SEM, ****p < 0.001, ANOVA with Tukey’s analysis. **d** Gene set enrichment analysis of GOBP regulation of cholesterol metabolic processes comparing LNCaP NTS vs LNCaP siMBTPS2 (*n* = 3 independent experiments, NES = −1.30). **e** Representative images of Filipin-based assay to detect cholesterol in LNCaP cells treated with NTS, siMBTPS2 or siSREBP siRNA. Cells were treated for 48 h and kept in either medium supplemented with cholesterol, or serum-free medium for 24 h prior to fixing. **f** Analysis of filipin staining (*n* = 3 independent experiments with >40 cells imaged per experiment, error bars are ±SEM, ***p < 0.005, ****p < 0.0001 ANOVA with Tukey’s analysis).

Gene set enrichment analysis (GSEA) identified that downregulated genes were enriched for fatty acid metabolism signalling following *MBTPS2* knockdown (Fig. 4a and Supplementary Fig. 6C). Consistent with the mechanism of reduced SREBP transcriptional activity, multiple SREBP target genes involved in fatty acid synthesis were altered, including ACACA and FASN modulation following *MBTPS2* silencing. Of particular interest, FASN, is known to catalyse the synthesis of long-chain saturated fatty acid such as palmitate from acetyl-CoA and malonyl-CoA. We performed immunocytochemistry to assay FASN levels following knockdown of either *MBTPS2* or *SREBP* in LNCaP cells (Fig. 4b). Suppressed MBTPS2 expression reduced FASN immunoreactivity by 40%. As expected, silencing of *SREBP* suppressed FASN expression by 60% (Fig. 4c).

**Fig. 5:**
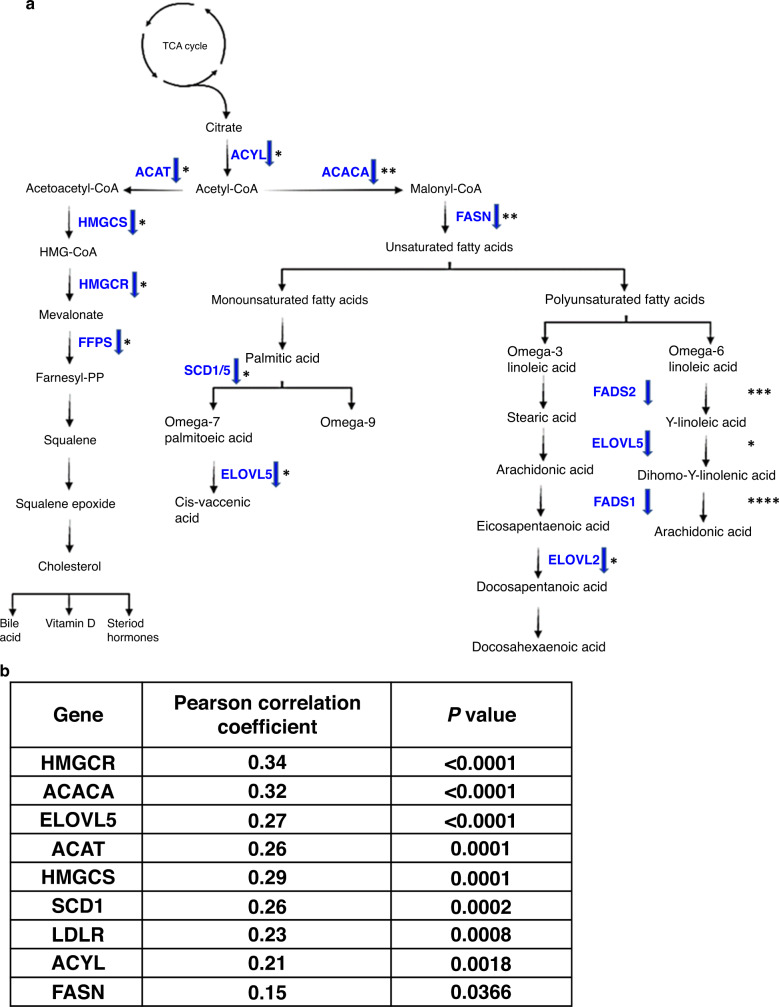
Knockdown of MBTPS2 and effects on key genes in cholesterol and fatty acid synthesis. **a** Differential expression of significant genes from RNA-Seq and/or qRT-PCR validation of RNA-Seq comparing LNCaP siMBTPS2 to LNCaP NTS. Blue arrow indicates decreased expression. ****P < 0.001, ***P < 0.005, **P < 0.01, *P < 0.05. **b** Correlation of *MBTPS2* expression with regulators of cholesterol and fatty acid synthesis genes from CBioPortal using SU2C/PCF Dream Team metastatic cohort(31).

GSEA also identified a reduction in regulators of cholesterol metabolism (Fig. 4d and Supplementary Fig. 6D). We manipulated LNCaP cells (with or without *MBTPS2* silencing) and their culture conditions (cholesterol-deficient medium or with free cholesterol supplement) and assayed the respective contents of free intracellular cholesterol (Fig. 4e, f). Upon silencing of *MBTPS2*, cholesterol levels were reduced by 38% (Fig. 4f) in cholesterol-free conditions. In cholesterol proficient growth condition, *MBTPS2* knockdown reduced intracellular cholesterol dramatically by 55% compared to NTS (Fig. 4f), suggesting that, besides *de novo* synthesis, cholesterol uptake may also contribute to its intracellular contents in a MBTPS2 dependent manner. Silencing of *SREBP* produced a similar effect to *MBTPS2* knockdown. (Fig. 4f). Consistent with the reported function of MBTPS2, silencing of *MBTPS2* in LNCaP cells significantly suppressed the expression of multiple SREBP target genes involved in biosynthesis and uptake of cholesterol and lipid: HMGCR 17%; LDLR 54%; ACACA 46%; and FASN 41% (Fig. 5a and Supplementary Fig. 7). HMGCR catalyses the conversion of HMG CoA to mevalonic acid and is known to be a rate-limiting enzyme in cholesterol biosynthesis [21]. Reduction in LDLR expression following *MBTPS2* loss may impair cellular uptake of cholesterol. Consistent with findings from the in vitro data, we were able to confirm the clinical relevance of *MBTPS2* in cholesterol and lipid metabolism using the SU2C/PCF Dream Team cohort (Fig. 2e, f). *MBTPS2* alterations also positively correlated with expression of key cholesterol and lipid biosynthesis genes (Fig. 5b).

Together, our in vivo screen and in vitro validation experiments implicate MBTPS2 to play a key role in progressive PC, likely via its regulation of cholesterol and fatty acid metabolism to regulate cellular proliferation.

## Discussion

The development of advanced and metastatic PC is dependent on changes in fatty acid and cholesterol metabolism [9, 22]. In particular, the SREBPs are the master regulators of numerous genes within both these pathways including FASN, HMGCR, ELOVLs and SCD [11].

Previous studies have demonstrated that SREBP1 is highly expressed in androgen-dependant PC and with androgen deprivation therapy (ADT) there is a significant reduction its levels, corresponding with clinical response. Critically, as the disease progresses, and there is a shift to “castrate-resistant” PC, and there is a re-emergence of high levels of SREBP1 [23]. Given that SREBPs coordinate the regulation of fatty acid and cholesterol metabolism gene expression we hypothesised that the changes of MBTPS2 could influence the SREBP transcriptional programme and subsequent development of PC [24].

We have demonstrated, using an unbiased forward mutagenesis screen, that mice which harboured insertions in the *Mbtps2* developed more locally advanced and metastatic prostate cancers compared to control mice (Fig. 2). Using clinical datasets (CBioPortal), we saw that up to 20% of metastatic PC had changes in either MBTPS2 or its downstream SREBF, with upregulation of these genes significantly reducing overall survival (Fig. 2). Subsequently, our in vitro data showed that knockdown of *MBTPS2* impairs human PC growth, proliferation, and invasion (Fig. 3). RNA-Seq of the *MBTPS2* knockdown cell lines demonstrated that changes in lipogenic signalling, and cholesterol metabolism could contribute to the cancer phenotype (Fig. 4). This corresponded with data from CBioPortal, where *MBTPS2* was found to positively correlate with several genes involved in lipid and cholesterol metabolism (Fig. 5b).

RNA-seq data also identified a reduction in the expression of Elongation of Very Long Chain Fatty Acids (ELOVL) protein family members. Elongation is a critical step in the production of fatty acids with a chain length of more than 16-carbons, and the ELOVL genes are critical in facilitating these reactions [25]. Our RNA-seq, and subsequent qPCR confirmed significant reductions in ELOVL5 expression in *MBTPS2*-knocked down LNCaP cells [26]. Additionally, ELOVL5 is known to be highly expressed in both primary and metastatic tumours, with numerous studies suggesting that downregulation of ELOVL5 has profound phenotypic effects on PC cells [27]. Interestingly, overexpression of MBTPS2 did not elevate growth of LNCaP or PC3 (Supplementary Fig. 4). As these PC cell lines have already increased expression of MBTPS2 compared to RWPE-1, this may indicate maximal pathway activation (i.e. SREBP cleavage) is achieved, and further MBTPS2 elevations are redundant. Alternatively, MBTPS2 levels may no longer be the rate-limiting step for SREBP cleavage, with the initial cleavage of S1P becoming the limiting step.

In this study, we have characterised the relationship of MBTPS2 and downstream lipid and cholesterol metabolism in progressive PC. Future studies would be required to investigate the role of S1P/MBTPS2-mediated ATF6 activation and subsequent ER-stress response and unfolded protein response. In summary, our findings are consistent with MBTPS2 influencing fatty acid and cholesterol synthesis to support prostate tumourigenesis. Further research should focus on validating the therapeutic potential of targeting MBTPS2 in advanced PC.

## Supplementary information

Supplementary Legends

Sup Figure 1

Sup Figure 2

Sup Figure 3

Sup Figure 4

Sup Figure 5

Sup Figure 6

Sup Figure 7

Sup Table 1

Sup Table 2

## Data Availability

The data that support the findings of this study are available on request from the corresponding author.
