# Supplementary material for: MBTPS2 acts as a regulator of lipogenesis and cholesterol synthesis through SREBP signalling in prostate cancer
Source: Br J Cancer. 2023 Mar 29;128(11):1991–9. doi: 10.1038/s41416-023-02237-7 (PMC10205813; doi:10.1038/s41416-023-02237-7)
Supplement: Supplementary file 1 — Supplementary Legends [file 41416_2023_2237_MOESM1_ESM.docx]

**Supplementary figure and table legends**

**Supplementary Figure 1**

Three representative H&E staining of murine prostate tumour samples at clinical endpoint of indicated genotypes*. Pten^Null^* tumours retain some glandular structure with evidence of crowding. *SB:Pten^Null^Mbtps2^WT^* show a less differentiated form of adenocarcinoma only slight remaining glandular architecture. *SB:Pten^Null^Mbtps2^INT^* appears largely undifferentiated with a dense tumour mass with no evidence of glandular structure.

**Supplementary Figure 2**

A) Co-occurrence of alterations in each of these indicated genes visualised on CBioPortal of SU2C/PCF Dream Team, PNAS 2019 cohort. B) Alteration data for each of MBTPS2 and AR genes.

**Supplementary Figure 3**

Rates of mutations shown for *MBTPS2* gene visualised on CBioPortal with indicated cohorts show with mutational characterising data indicated. Green indicates mutation by missense or truncation, red indicates copy number amplification, blue indicates deep deletion of gene. Each study reference is given in the figure.

**Supplementary Figure 4**

**A.** Immunofluorescence assay from LNCaP and PC3 cells treated with MBTPS2 overexpressing plasmid in comparison to mock treated control including representative images of each condition. **B.** *Mbtps2* expression as quantified by qPCR in indicated cell lines treated with mock transfection or MBTPS2 overexpressing plasmid. **C.** Invasion assay from LNCaP and PC3 cells treated with MBTPS2 overexpressing plasmid in comparison to mock treated control**.** Data presented as % change in invasion relative to control. **D.**  Growth analysis data from LNCaP and PC3 cells treated with MBTPS2 overexpressing plasmid (red) in comparison to mock treated control (black). Cells were grown in either 2D or 3D culture as indicated.

**Supplementary Figure 5**

A) qPCR relative quantification (RQ) data demonstrating the mRNA levels of *MBTPS2* after siRNA targeted treatment for either *MBTPS2* or NTS in LNCAP cells to validate knockdown prior to RNA-Seq (n=3 independent experiments, error bars are ± SEM, **p<0.01, Mann-Whitney. B) Principle component analysis (PCA) demonstrating variance between RNA-Seq samples of LNCAP cells treated with either NTS (red) or siMBTPS2 (blue) from different experiments (n=3 experiments). C) Heatmap showing Z-score of genes from RNA-Seq of LNCAP treated with either NTS (red) or siMBTPS2 (blue). Blue z-score indicates reduction in gene expression, green indicates no change in gene expression, yellow indicating increase in gene expression relative to other group. D) Volcano plot showing fold change and significance of gene expression between RNA-Seq samples of LNCaP cells treated with either NTS or siMBTPS2. Genes were filtered based on a fold change >1.5 and p-value <0.05.

**Supplementary Figure 6**

A-B) Upregulated and downregulated networks from RNA-Seq comparing LNCAP NTS vs LNCAP siMBTPS2 (n=3 independent experiments). Visualised using MetaCore, genes filtered by p<0.05 and fold change >1.5. C-D) Geneset enrichment analysis of GOBP fatty acid metabolic process and GOBP regulation of cholesterol esterification comparing LNCaP NTS vs LNCaP siMBTPS2 (n=3 independent experiments, normalised enrichment score (NES) = -1.51 and -1.39 respectively).

**Supplementary Figure 7**

qPCR data showing the mRNA levels of a panel of cholesterol and lipid synthesis genes after siRNA targeted treatment for either *MBTPS2* or NTS in LNCaP cells, respectively (n=3 independent experiments, error bars are ±SEM,*p<0.05, **p<0.01, ****p<0.0001, ANOVA with Tukey’s analysis. (*HMGCS*; HMG-CoA synthase, *HMGCR*; HMG-CoA reductase, Acetyl-CoA carboxylase 1; *ACACA*, Fatty acid synthase; *FASN*, Low density lipoprotein receptor; *LDLR*, SCD1; Stearoyl-CoA Desaturase 1)

**Supplementary Table 1**

Targeting and non-targeting siRNA sequences and their corresponding catalogue numbers.

**Supplementary Table 2**

QPCR primer sequences and their target gene of interest, including Rocha Universal Probe for detection and corresponding catalogue numbers.
