# Supplementary figures and images for: MBTPS2 acts as a regulator of lipogenesis and cholesterol synthesis through SREBP signalling in prostate cancer
Source: Br J Cancer. 2023 Mar 29;128(11):1991–9. doi: 10.1038/s41416-023-02237-7 (PMC10205813; doi:10.1038/s41416-023-02237-7)

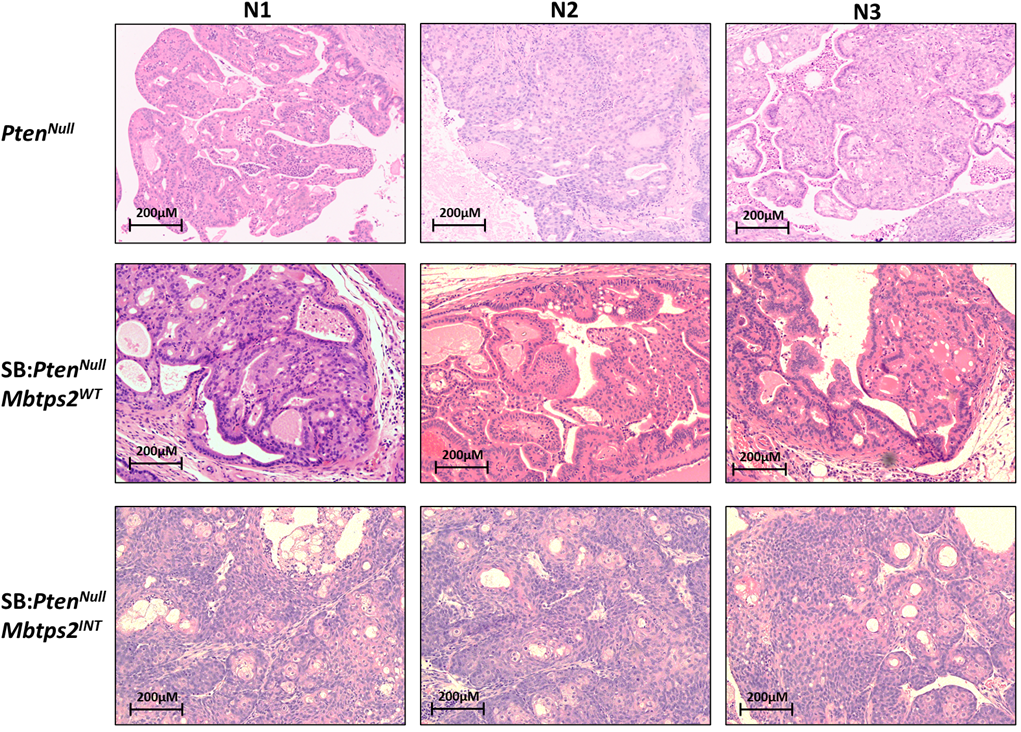

Supplement: Supplementary file 2 — Sup Figure 1 [file 41416_2023_2237_MOESM2_ESM.png]

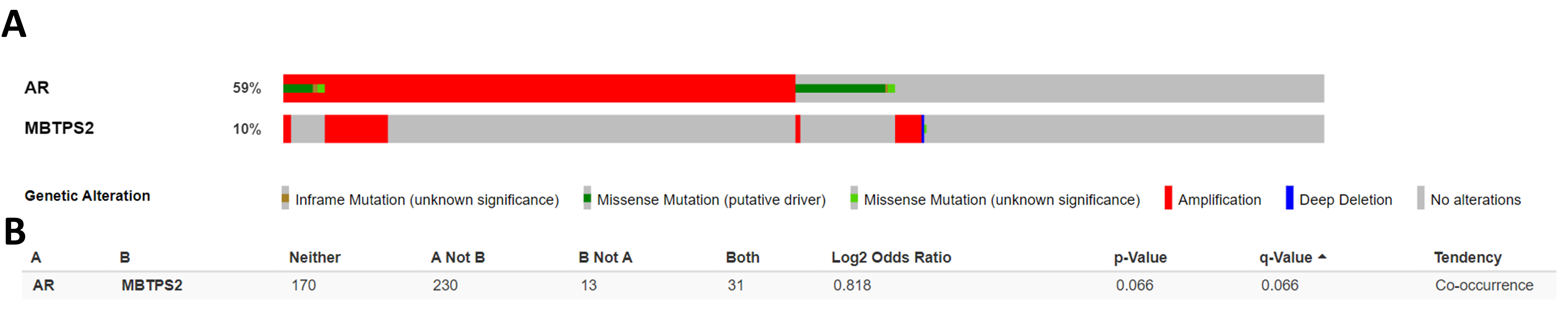

Supplement: Supplementary file 3 — Sup Figure 2 [file 41416_2023_2237_MOESM3_ESM.png]

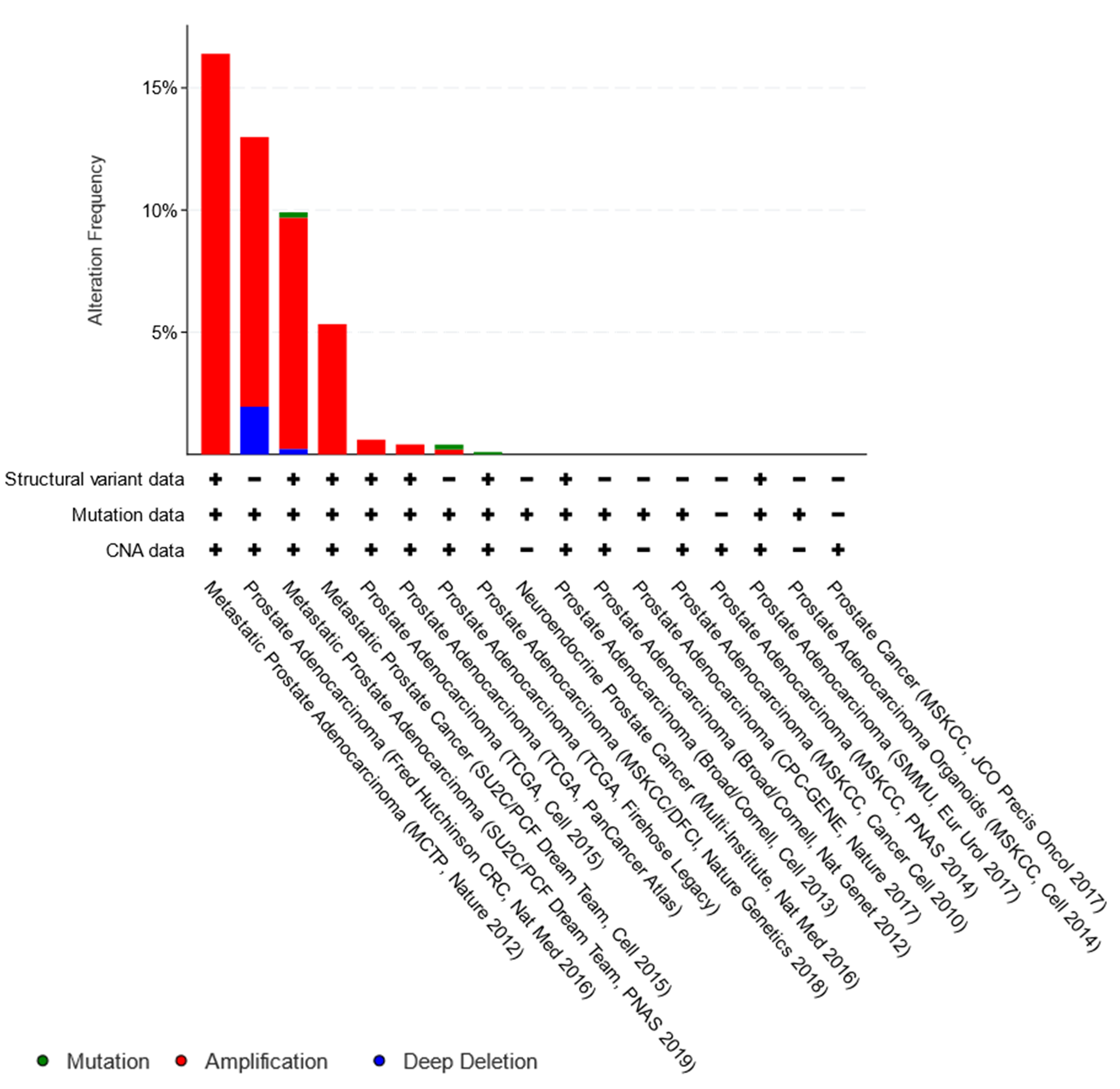

Supplement: Supplementary file 4 — Sup Figure 3 [file 41416_2023_2237_MOESM4_ESM.png]

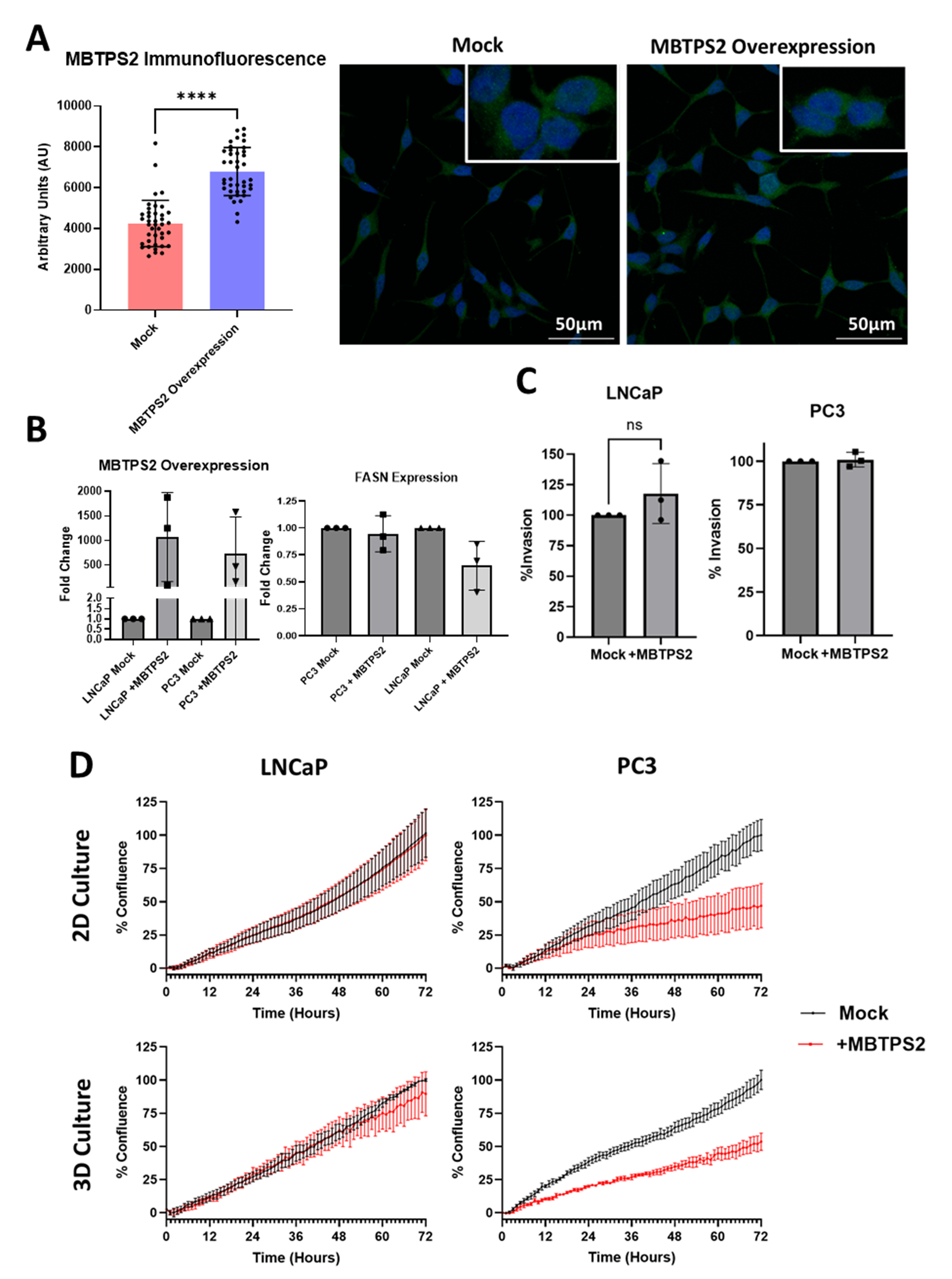

Supplement: Supplementary file 5 — Sup Figure 4 [file 41416_2023_2237_MOESM5_ESM.png]

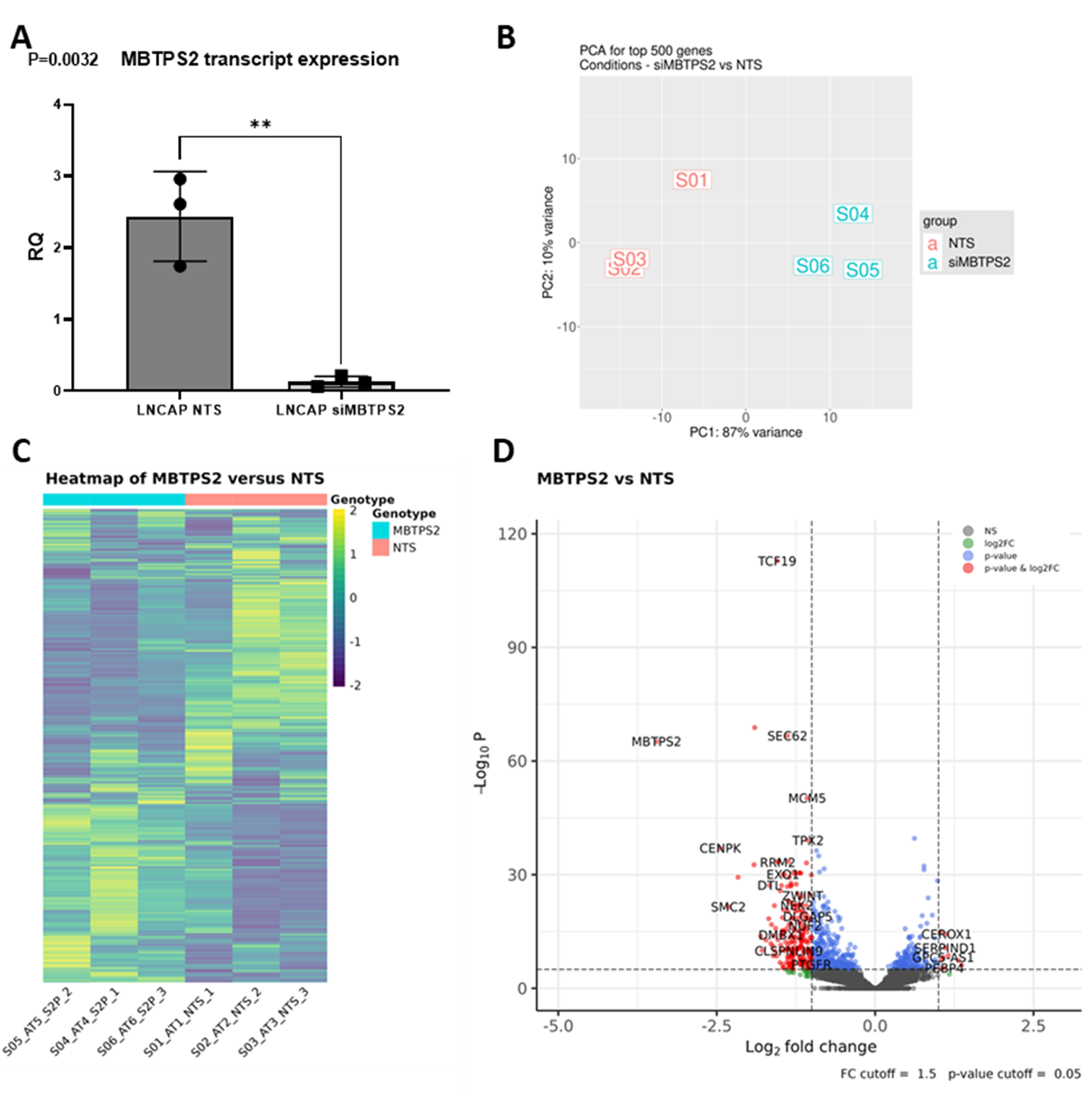

Supplement: Supplementary file 6 — Sup Figure 5 [file 41416_2023_2237_MOESM6_ESM.png]

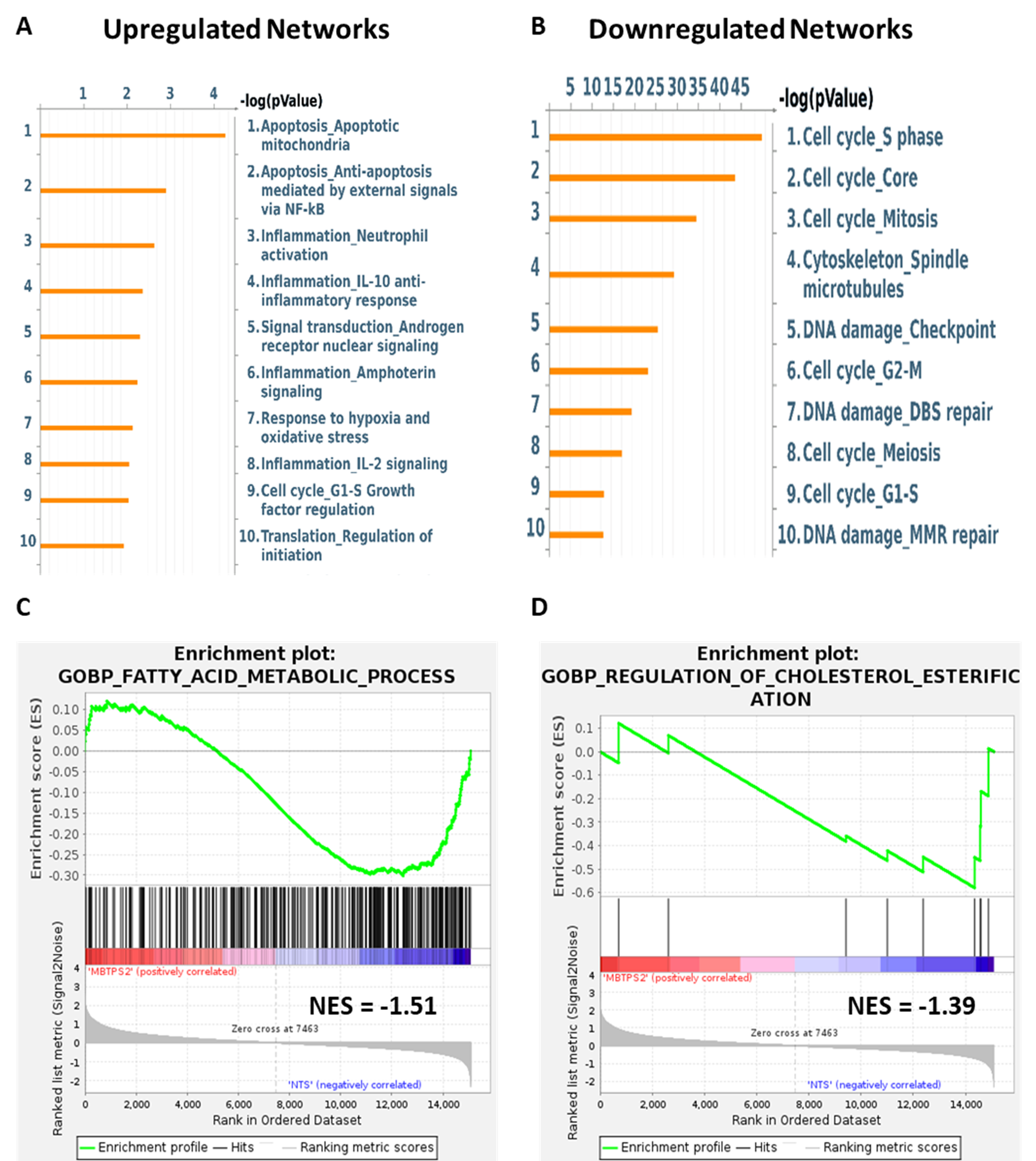

Supplement: Supplementary file 7 — Sup Figure 6 [file 41416_2023_2237_MOESM7_ESM.png]

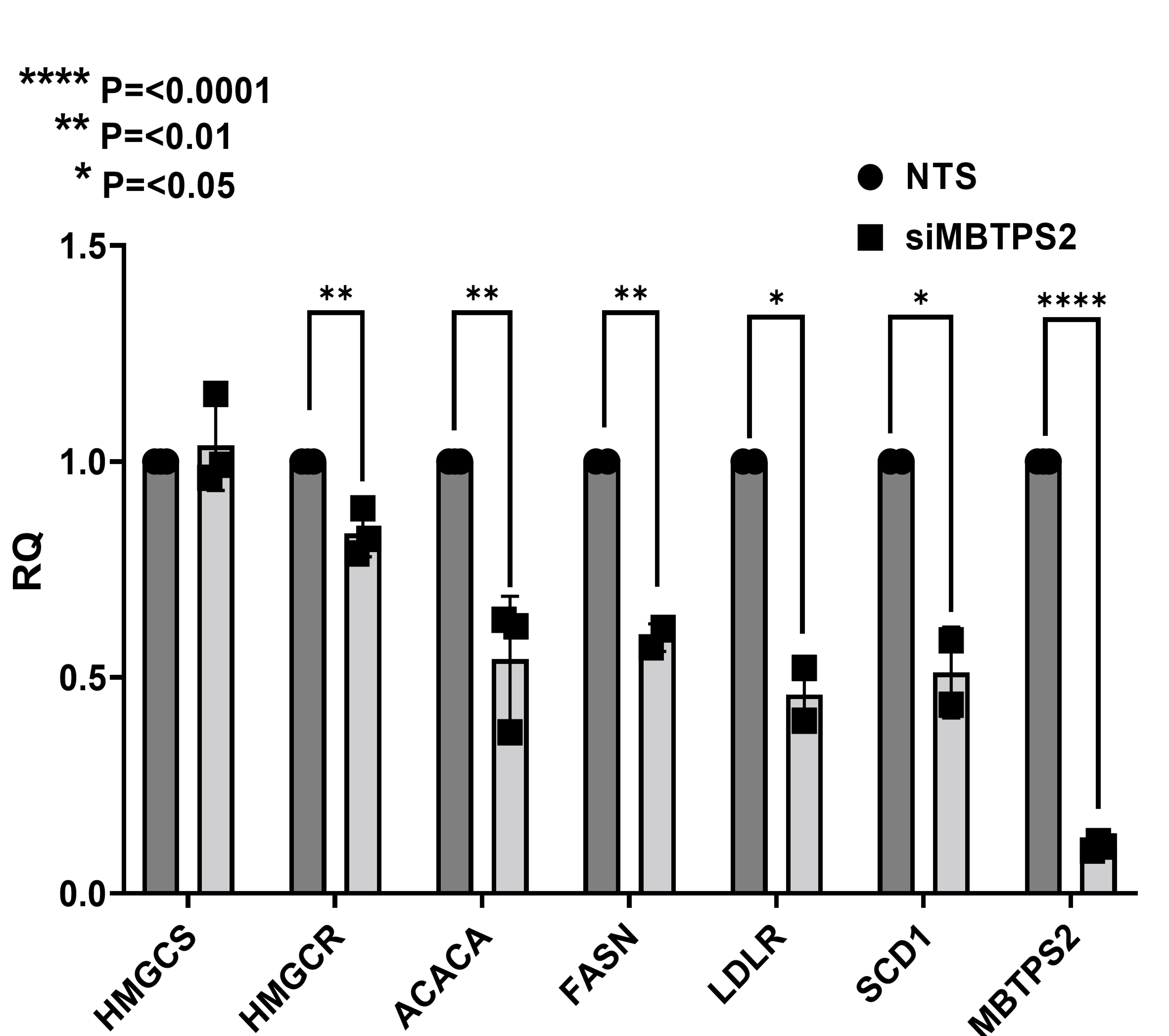

Supplement: Supplementary file 8 — Sup Figure 7 [file 41416_2023_2237_MOESM8_ESM.png]

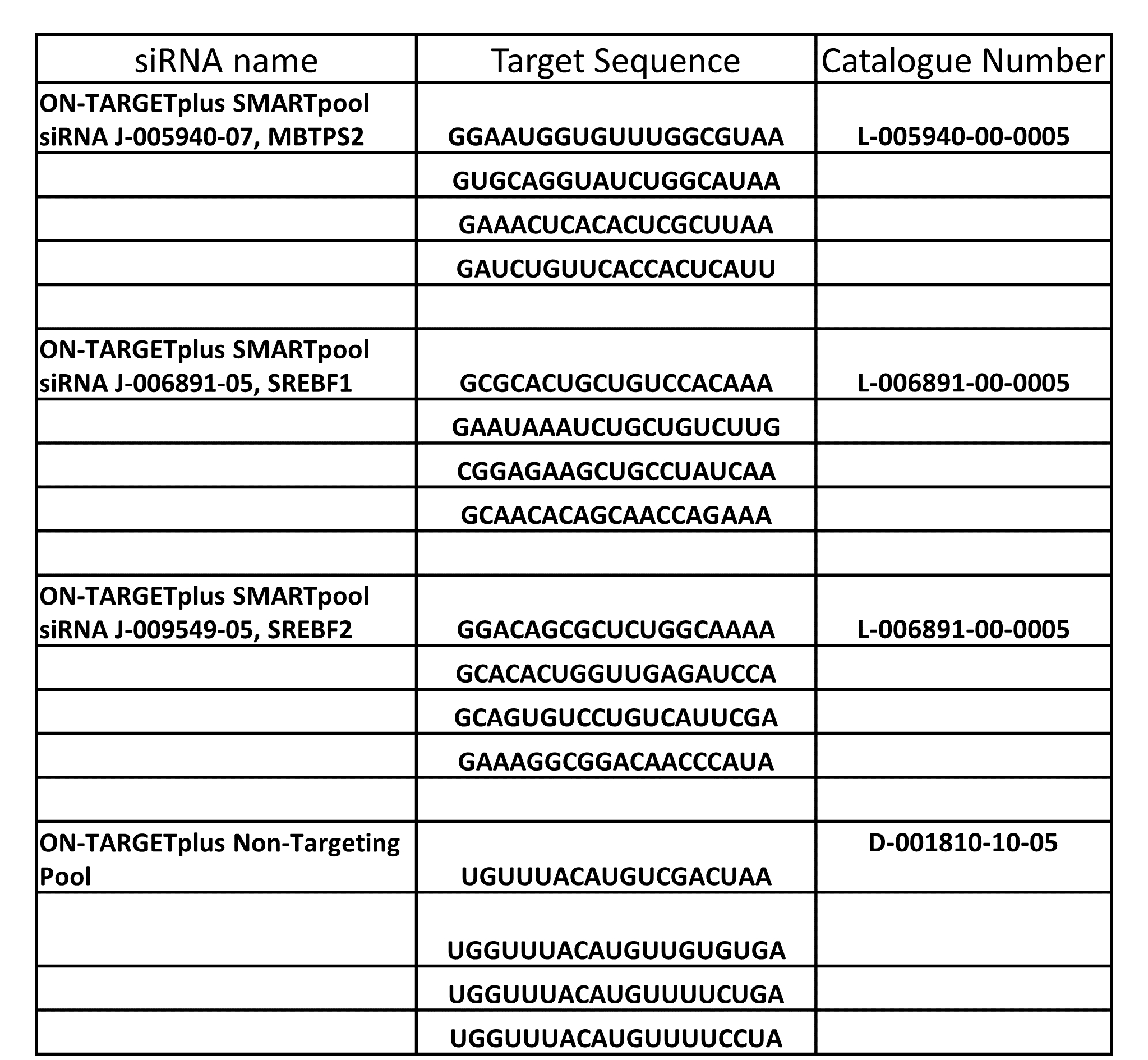

Supplement: Supplementary file 9 — Sup Table 1 [file 41416_2023_2237_MOESM9_ESM.png]

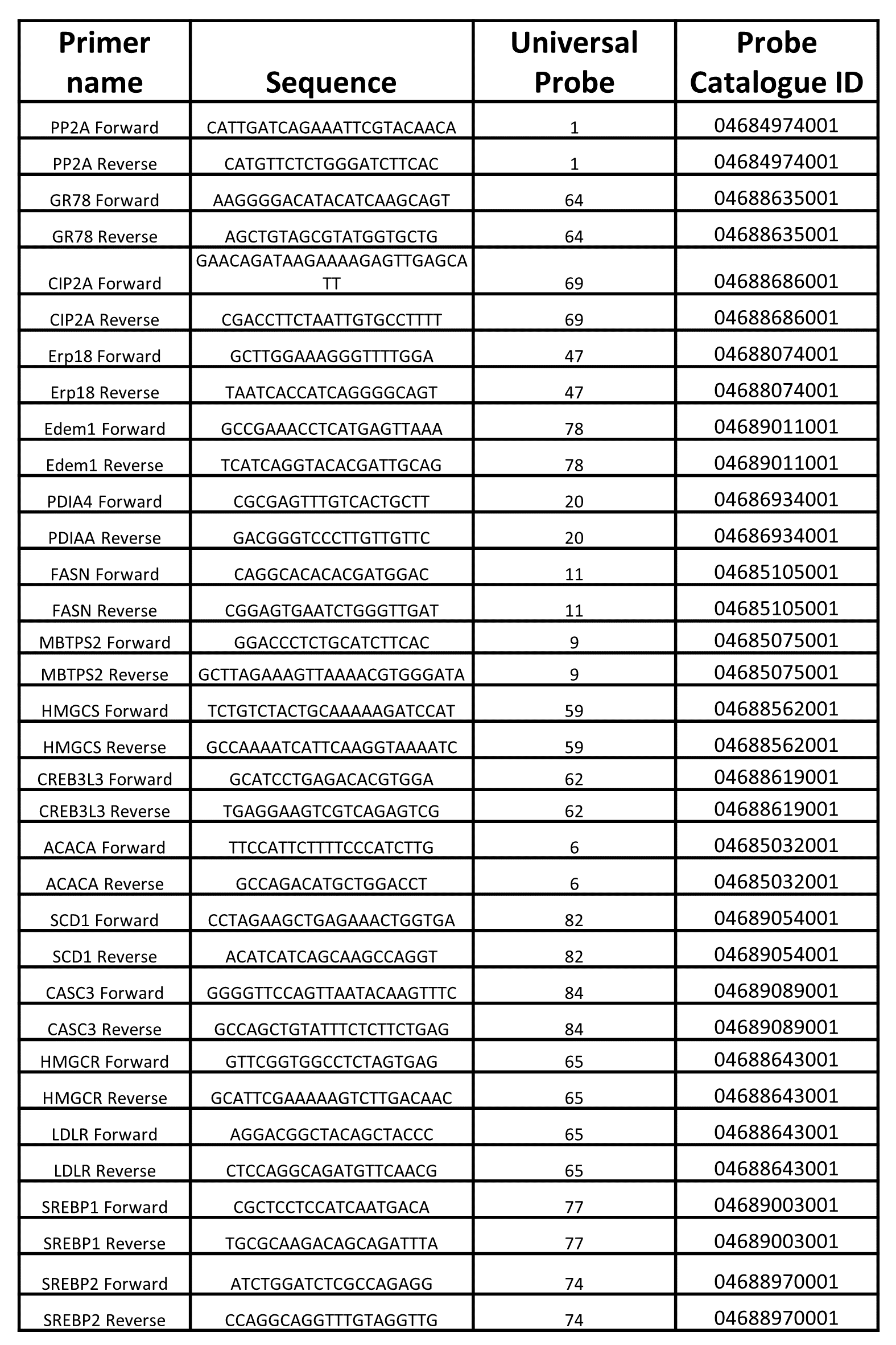

Supplement: Supplementary file 10 — Sup Table 2 [file 41416_2023_2237_MOESM10_ESM.png]
